# Letter from Surgeon-General J. B. Hamilton to Medical Officers

**Published:** 1879-07

**Authors:** J. B. Hamilton, John Sherman

**Affiliations:** Surgeon-General U. S. Marine-Hospital Service; Washington, D. C.; Secretary of the Treasury; Washington, D. C.


TREASURY DEPARTMENT,
OFFICE SUP. SURG.-GEN. U. S. MARINE-HOSPITAL SERVICE,
WASHINGTON, D. C., June 11, 1879.

*To the Medical Officers of the Marine-Hospital Service,*
*And all others whom it may concern:*

1. To insure to such owners of American vessels as desire the services of sound and healthy seamen, facilities for the proper physical examination of crews, at all ports where medical officers of the Marine-Hospital Service are stationed, such officers will, upon the application of any U. S. Shipping Commissioner, or of the master or owner of any vessel engaged in the foreign trade, or passenger steamer engaged in the coasting trade, examine physically any seaman or seamen, and give a certificate as to their fitness or otherwise.

2. A record will be kept of all examinations of seamen, and a transcript thereof forwarded quarterly to the Surgeon-General of the Marine-Hospital Service.

3. In all cases of rejection, the certificate will state explicitly, in English, the reason for such rejection.

4. The loss of an arm or leg; defective vision; color blindness; epilepsy; mental unsoundness; hernia; piles; fistulæ; varicose veins; serious organic diseases; habitual drunkenness; the existence of venereal disease; marked want of development; weakness of the body, or deformity; should cause the rejection of any seaman desiring to ship.

5. No seaman will be examined for the purpose of giving such certificate except in the presence of a United States Shipping Commissioner, or the master, owner, or agent of the vessel on which the seaman is expected to be employed, and examinations will only be made at the Marine-Hospital Office.

6. The rejection of a seaman at one examination shall not debar him from subsequent examination in case he claims that the disease for which he was rejected has disappeared.

7. The provisions of this circular will also apply to enlisted persons in the Revenue-Marine, Life-Saving, Coast-Survey, and Light-

House Services, and to persons desiring to enlist therein, upon the application of the proper officers of the respective ser vices.

8. No fee will be charged by any medical officer for making the examination or certificate herein contemplated.

J. B. HAMILTON,
Surgeon-General U. S. Marine-Hospital Service.

APPROVED:

JOHN SHERMAN,

Secretary of the Treasury.

